# Correlation of Hemoglobin A1c with Red Cell Width Distribution and Other Parameters of Red Blood Cells in Type II Diabetes Mellitus

**DOI:** 10.7759/cureus.5533

**Published:** 2019-08-30

**Authors:** Abdul Rabb Bhutto, Amanullah Abbasi, Ali Hassan Abro

**Affiliations:** 1 Internal Medicine, Al-Tibri Medical College & Hospital Isra University Karachi Campus, Karachi, PAK; 2 Internal Medicine, Dow University of Health Sciences, Karachi, PAK; 3 Internal Medicine, Southend University Hospital, Southend, GBR

**Keywords:** rdw, hba1c, diabetes mellitus

## Abstract

Objective

To determine the correlation of glycated hemoglobin (HbA1c) with red cell width (RDW) and other analytic parameters of red blood cells (RBCs) in type II diabetic patients.

Design

Cross-sectional analytical study.

Place and duration of the study

Al-Tibri Medical College and Hospital Karachi; from July 2017 to January 2018.

Patients and methods

This cross-sectional study was conducted on diagnosed type II diabetic patients visiting the outpatient department of medicine at Al-Tibri Medical College Hospital from July 2017 to January 2018. Diabetes mellitus was diagnosed according to American Diabetes Association (ADA) guidelines. After taking consent and conducting a clinical assessment (include history and physical examination), laboratory tests, such as fasting blood glucose, random blood glucose, complete blood count (CBC), and HbA1c, were collected on proforma.

Results

A total of 119 patients were eligible for the study with a mean age of 48.63±12.462 (range 24-76) years; among those, males were 74 (62.2%) and females were 45 (37.8%). The mean duration of diabetes mellitus (DM) was 6.735±3.759 (range 1-20) years. The mean hemoglobin of patients was 11.59±1.315 gm/dl. The mean corpuscular volume (MCV) was 76.65±11.121 fl and the mean RDW was found to be 18.287±4.352, with the highest value of 30.20. The mean MCH was 30.223±23.873 pg, with the highest value of 38.4 pg. The mean cell hemoglobin concentration (MCHC) was 28.214±4.7498 mg/dl.

The HbA1c of the study population was found to be moderately uncontrolled and the mean HbA1c was 8.278±5.015%, with the highest value of 16.2%. The mean fasting blood sugar was 158±39.50 mg/dl while the mean random blood sugar was 236±57.390 mg/dl.

The correlation of HbA1c with RDW turned out to be significant statistically (p-0.035) while other RBCs and/or hematological parameters, such as MCV, hemoglobin, and platelets, revealed no significant correlation.

Conclusion

The study highlighted that RDW has a significant correlation with HbA1c and is an inexpensive and freely available test so it may be used as a marker of glycemic status.

## Introduction

Globally, it is estimated that there are 382-million people living with diabetes, and the International Diabetes Federation has projected that 592-million people will have diabetes by 2035 [1]. The rising incidence and chronic nature lead to an increased risk of a complex complication profile of macrovascular and microvascular complications. Hence, following a diagnosis of diabetes, management, regular follow-up with an assessment for complications, and blood sugar monitoring always remain a big challenge for any health care provider, especially in low socioeconomic countries. The cost-effectiveness of those measures or tools for the assessment of complications status and blood sugar monitoring is highly desirable and encouraged. In this regard, recently, glycated hemoglobin (HbA1c) was used as a tool for glycemic control but since 2010, it is now accepted for the diagnosis of diabetes too [2]. The HbA1c concentration depends on the plasma glucose concentration and the duration of hyperglycemia. HbA1c concentration is measured as an index of glycemic control for a period over eight to 12 weeks.

Besides the glycosylation of hemoglobin, hyperglycemia has several other effects on red blood cells (RBCs) as well as changes in the mechanical properties of RBCs, reduced deformability, increased adhesion, and increased osmotic fragility, consequently leading to changes in erythrocyte structure and hemodynamic characteristics [3-4].

In recent years, the diagnostic and prognostic value of a parameter of RBCs, red cell distribution width (RDW), has been studied in various diseases like cardiovascular mortality [5] and peripheral artery disease [6]. RDW is a measure of variability in the size of RBCs and is a component of routine complete blood counts (CBCs). Traditionally, it is used together with the mean corpuscular volume (MCV) in clinical practice to differentiate between the causes of anemia [7].

Uncontrolled diabetes leading to the consistent elevation of HbA1c may induce functional and structural changes in the hemoglobin molecule and cytoplasmic environment within each red blood cell. Consequently, the RDW and other RBC parameters may be altered. As the microvascular complications of diabetes mellitus are well-proven and linked to increased HbA1c, alteration in RDW might have a role in the diagnosis and monitoring of glycemic status as well as complication assessment in diabetic patients. The aim of this study was to assess the correlation between HbA1c and RDW, as it is more cost-effective, routinely a component of CBC.

## Materials and methods

Patients and methods

This cross-sectional analytical study was conducted on diagnosed type II diabetic patients visiting the outpatient department of medicine at Al-Tibri Medical College Hospital from July 2017 to January 2018. All patients provided written consent for data collection, which was approved by our institutional ethical committee. Diabetes mellitus was diagnosed according to American Diabetic Association (ADA) guidelines: HbA1c ≥ 6.5% or fasting blood glucose (FBG) ≥ 126 mg/dl or two-hour plasma glucose ≥ 200 mg/dl during an oral glucose tolerance test (OGTT).

Patients were excluded from the study if they had a history of hemoglobinopathies, anemia of any cause, chronic illnesses like chronic liver disease, chronic renal failure, rheumatologic disorders, and acute or chronic infections like malaria, tuberculosis, or malignancy. After taking consent, all the subjects underwent a detailed history, thorough physical examination, and routine relevant laboratory investigations for the implementation of inclusion and exclusion criteria.

The hematology analyzer - Nihon-Kohden Celltac-a (Tokyo, Japan) was used for blood parameters measurement while HbA1c was analyzed by the hemoglobin analyzer - GP Getein 1100 ( Getein Biotech, Inc., China) 

Fasting blood glucose, random blood glucose, CBC, and HbA1c were collected on proforma.

Statistical analysis

Data were analyzed by using SPSS 23 (Statistical Package for Scientific Studies; IBM Corp., Armonk, NY, US) for Windows. Data were described as mean and standard deviation (SD). Correlation between different variables was done using the Pearson moment correlation equation for a linear relation in normally distributed variables. A p-value of less than or equal to 0.05 was considered statistically significant.

## Results

A total of 119 patients was found eligible for the study, with a mean age of 48.63±12.462 (range 24-76) years, and out of those 119 patients, males were 74 (62.2%) and females were 45 (37.8%). On the basis of ethnicity, 54 (45.4%) were Sindhi followed by Balochi 28 23.55), Pakhtoon 24 (202%), Urdu 11 (9.2%), and Punjabi 02 (1.7%). According to marital status, the majority of the study population (92.4%) was married. As per the educational status of the study population, 32 (26.9%) subjects were either illiterate or only had incomplete Deeni/Madarsa education, 21 (17.6%) had a primary level of education, 26 (21.8%) had passed middle school, 27 (22.7%) had completed matriculation, 12 (10.1%) had finished intermediate school while only one (0.8%) subject had graduated. The baseline demographics of the study subjects are shown in Table 1.

**Table 1:** Baseline demographics of study population (n-119)

Variable	Frequency	Percentage
Gender		
Male	74	62.2
Female	45	37.8
Age (years)		
Less than or equal 40	36	30.3
More than 40	83	69.7
Marital Status		
Single	09	7.6
Married	110	92.4
Educational status		
Deeni/Madarsa	32	26.9
Primary	21	17.6
Middle	26	21.8
Matric	27	22.7
Intermediate	12	10.1
Graduate	01	0.8
Language		
Balochi	28	23.5
Pakhtoon	24	20.2
Punjabi	02	1.7
Sindhi	54	45.4
Urdu	11	9.2
Total	119	100

The mean duration of DM was 6.735±3.759 (range 1-20) years. The mean hemoglobin of patients was 11.59±1.315 gm/dl. MCV was 76.65±11.121 fl and mean RDW was found to be 18.287±4.352, with the highest value of 30.20. MCH was 30.223±23.873 pg, with the highest value of 38.4 pg. MCHC was 28.214±4.7498 mg/dl. The HbA1c of the study population was found moderately uncontrolled and the mean HbA1c was 8.278±5.015%, with the highest value of 16.2%. The mean fasting blood sugar was 158±39.50 mg/dl while the mean random blood sugar was 236±57.390 mg/dl.

The correlation of HbA1c with RDW turned out to be significant statistically (p-0.035) while other RBCs and/or hematological parameters like MCV, hemoglobin, and platelets revealed no significant correlation, as shown in Table 2.

**Table 2:** Correlation of HbA1c with different hematological parameters (n=119) MCV: Mean corpuscular volume; MCH: Mean corpuscular hemoglobin; MCHC: Mean corpuscular hemoglobin concentration; RDW: Red blood cell distribution width; FBS: Fasting blood sugar; RBS: Random blood sugar; WBC: White blood cells

Parameter	Correlation	P-value
Hemoglobin	0.098	0.290
MCV	-0.127	0.167
MCH	-0.109	0.238
MCHC	0.051	0.583
RDW	0.193	0.035*
FBS	0.324	<0.0001*
RBS	0.388	<0.0001*
WBC	-0.025	0.869
Platelets	0.108	0.244

## Discussion

Diabetes mellitus is a metabolic disease secondary to either the deficiency or decreased responsiveness of tissues to insulin and is associated with derangements in the metabolism of carbohydrates, lipids, and proteins. Hence, insulin is a very important hormone in metabolic homeostasis. Persistent hyperglycemia resulting from insulin deficiency leads to the devastating and life-threatening complications of diabetes. Keeping this fact in mind, frequent monitoring of blood sugar is a key step in the management of diabetes. Traditionally, fasting and random or postprandial blood sugar have been used in glycemic monitoring. In recent years, HbA1c has been added as a tool to diagnose or monitor diabetes as well as play a role in predicting the complications, mortality, and morbidity of diabetes mellitus.

Persistently elevated plasma glucose is nonenzymatically glycated to hemoglobin A to form HbA1c, hence, the concentration of HbA1c depends on the level and duration of glucose in the plasma that is measured clinically as an index of diabetic control for a period over eight to 12 weeks.

Moreover, like HbA1c, RDW is also one of the parameters of RBCs, but it is reported along with a simple routine laboratory investigation of the CBC without any extra cost and can be defined as a marker of the variation in cell volume within the red cell population that is reported as an index of heterogeneity in the size of circulating erythrocytes [8]. RDW has been extensively studied in various studies on the etiology and diagnosis of anemia [9]. More recently, its diagnostic and prognostic role has been discovered in various diseases and their adverse outcomes have been associated with high RDW, such as increased mortality [10], bad prognostic marker in heart failure or ischemic heart disease patients [11-12], and a higher incidence of atrial fibrillation and heart failure [13-14].

In our study, we found a statistically significant correlation of RDW with HbA1c (r=0.193, p 0.035), and the same observations were evidenced by Suryavanshi et al. in their study (r=-0.235, p=0.001) [15] while Salimon et al. found in their study that males have shown a significant correlation comparatively more than females (r= 0.400 vs r= 0.04) [16]. Another study by Sherif et al. has also shown a positive correlation between RDW and HbA1c but, statistically, it was not significant (p=092) [17]. Moreover, Lippi et al. also demonstrated a significant correlation of HbA1c and RDW in their study even after adjustment for age and gender [18].

While other parameters of RBCs like MCV, MCH, and MCHC have shown no significant correlation with HbA1c. There is diversity in the results of previous studies regarding the correlation of these parameters and HbA1c; a study by Hardikar et al. on non-diabetic subjects observed an inverse correlation between HbA1c and MCV (r = -0.22, p < 0.05), MCH (r = -0.30, p < 0.05), and MCHC (r = -0.32, p < 0.05) [19] while another study by Koga et al. found HbA1c was inversely associated with MCV (r = -0.368, p < 0.0001) and MCH (r = -0.320, p < 0.0001) in premenopausal women but postmenopausal women have shown no such relation between HbA1c and MCV (r = -0.019, p = 0.771) and MCH (r = -0.104, p = 0.107) [20].

There were a few limitations to our study. First, this study was a single-center-based study with a limited sample so could not be a representation of the whole population of the country; second, the study was conducted based on duration, so the sample size was not calculated.

## Conclusions

As it is a well-known fact that diabetes mellitus is a life-long metabolic disease, patients with DM keep asking for cost-effective and easily available means of monitoring their glycemic status. In that context, our current study highlighted that RDW has a significant correlation with HbA1c and is an inexpensive and freely available test. Therefore, it may be used as a marker of glycemic status. However, further studies on a larger scale are required to detect this relation and its glycemic monitoring role in diabetic patients.
